# Cholinergic Enhancement of Cell Proliferation in the Postnatal Neurogenic Niche of the Mammalian Spinal Cord

**DOI:** 10.1002/stem.2077

**Published:** 2015-06-26

**Authors:** Laura F. Corns, Lucy Atkinson, Jill Daniel, Ian J. Edwards, Lauryn New, Jim Deuchars, Susan A. Deuchars

**Affiliations:** ^1^ School of Biomedical SciencesUniversity of LeedsLeedsUnited Kingdom

**Keywords:** Acetylcholine, Cell proliferation, Ependymal, Spinal cord, Neurogenic niche

## Abstract

The region surrounding the central canal (CC) of the spinal cord is a highly plastic area, defined as a postnatal neurogenic niche. Within this region are ependymal cells that can proliferate and differentiate to form new astrocytes and oligodendrocytes following injury and cerebrospinal fluid contacting cells (CSFcCs). The specific environmental conditions, including the modulation by neurotransmitters that influence these cells and their ability to proliferate, are unknown. Here, we show that acetylcholine promotes the proliferation of ependymal cells in mice under both *in vitro* and in vivo conditions. Using whole cell patch clamp in acute spinal cord slices, acetylcholine directly depolarized ependymal cells and CSFcCs. Antagonism by specific nicotinic acetylcholine receptor (nAChR) antagonists or potentiation by the α7 containing nAChR (α7*nAChR) modulator PNU 120596 revealed that both α7*nAChRs and non‐α7*nAChRs mediated the cholinergic responses. Using the nucleoside analogue EdU (5‐ethynyl‐2'‐deoxyuridine) as a marker of cell proliferation, application of α7*nAChR modulators in spinal cord cultures or in vivo induced proliferation in the CC region, producing Sox‐2 expressing ependymal cells. Proliferation also increased in the white and grey matter. PNU 120596 administration also increased the proportion of cells coexpressing oligodendrocyte markers. Thus, variation in the availability of acetylcholine can modulate the rate of proliferation of cells in the ependymal cell layer and white and grey matter through α7*nAChRs. This study highlights the need for further investigation into how neurotransmitters regulate the response of the spinal cord to injury or during aging. Stem Cells *2015;33:2864–2876*

**Significance Statement**

Our work has shown that by, activating a specific neurotransmitter receptor, we can transform a population of spinal cord cells, the ependymal cells from being a quiescent stem cell population into one that can proliferate and thus enable spinal cord plasticity. Our findings are therefore of wide scientific interest to those involved in understanding adult neurogenesis and spinal cord function in health and disease. Perhaps most critically, these data will be of interest to those who are seeking to understand how we can harness the body's natural ability to trigger cell proliferation and differentiation or even to suppress inappropriate proliferative responses.

## Introduction

In the mammalian spinal cord, the area surrounding the central canal (CC) exhibits plasticity. First, ependymal cells display neural stem cell features *in vitro* as they are capable of neurosphere formation, self‐renewal and differentiation into the three neural lineages 1, 2. In vivo, spinal cord ependymal cells can proliferate under both normal and injured conditions and differentiate into astrocytes and oligodendrocytes following injury 3, 4, 5 and into neurons in a model of multiple sclerosis 6.

Second, there is a population of cells found either in the subependymal layer or interspersed with ependymal cells which possess a large cerebrospinal fluid‐contacting process; thus, they are known as cerebrospinal fluid contacting cells (CSFcCs). CSFcCs express immunohistochemical markers of immature neurons or neurons involved in plasticity, such as Doublecortin, PSA‐NCAM (polysialylated neuronal cell adhesion molecule) and growth‐associated protein 43 7, 8 and display electrophysiological properties consistent with them being neurons at different stages of maturity 7. Their proliferative capability is less well understood.

Considering the stem cell potential of ependymal cells, understanding physiological factors influencing their function and manipulating their proliferation rate could provide an avenue to replenish spinal cord cellular pools that are depleted by injury or disease. Such a physiological factor, endogenous dopamine, promotes the generation of spinal motor neurons in lesioned adult zebrafish spinal cord 9.

Spinal cord regeneration in mammals is less successful and the influence of neurotransmitters is unknown; therefore, we determined whether CSFcCs and/or ependymal cells were modulated by neurotransmitters, with a focus on acetylcholine (ACh). Neurotransmitters, including ACh, can regulate proliferation, neuronal differentiation and maturation in established areas of mammalian postnatal neurogenesis such as the subventricular zone of the forebrain and the subgranular zone in the hippocampal dentate gyrus 10. For example, reducing cholinergic inputs to the olfactory bulb decreases numbers of newly born neurons 11, while their survival was improved by enhancing cholinergic signaling using the centrally acting reversible acetylcholinesterase inhibitor, donepezil 12. In the dentate gyrus, newborn cells receive cholinergic inputs 13 and the normal survival, maturation, and integration of adult‐born neurons is reliant on functional α7‐containing nicotinic acetylcholine receptors (α7*nAChRs 14). In the subventricular zone, cholinergic neurons have recently been identified that enhance neural stem cell proliferation, thus cholinergic circuits may be critical during numerous stages of postnatal neurogenesis 15. Cholinergic interneurons reside in the CC region of the spinal cord 16, 17 and thus are well placed to influence cells in the neurogenic niche.

Given the influence of ACh on cells in other postnatal neurogenic niches and the neural stem cell potential of ependymal cells, we hypothesized that these cells are influenced by activation of cholinergic receptors which modulate their proliferative capacity. We use electrophysiology in an in vitro spinal cord slice preparation to demonstrate that both ependymal cells and CSFcCs respond to ACh. Furthermore, applications of a cholinergic modulator *in vitro* to organotypic slice cultures and in vivo combined with 5‐ethynyl‐2'‐deoxyuridine (EdU) labeling reveal that cholinergic stimulation enhances the proliferation of ependymal cells, but not CSFcCs.

## Materials and Methods

Full details are given in Supporting Information.

### Animals

Wistar rats (P9‐adult) or C57/Bl6 mice (P9‐adult) of either sex were used in line with the UK Animals (Scientific Procedures) Act 1986 and ethical standards set out by the University of Leeds Ethical Review Committee. Every effort was made to minimize the number of animals used and their suffering.

### Slice Preparation

Animals (9–28 days) were anaesthetized with sodium pentobarbitone (60 mg/kg) I.P and perfused transcardially with ice‐cold sucrose artificial CSF (aCSF) before cutting 300 µm thick transverse spinal cord slices using a vibrating microtome.

### Whole Cell Patch Clamp Electrophysiology

Whole cell current clamp recordings were made at room temperature from CSFcCs and ependymal cells. Neurobiotin (0.5 %; Vector Laboratories, Peterborough, UK, https://www.vectorlabs.com/uk/) and tetramethylrodamine (0.02 %; Life Technologies, Paisley, UK, https://www.lifetechnologies.com/uk/en/home.html) were added to visualize the cells post‐recording (see ref. 18).

ACh (3–10 mM) was pressure ejected locally; cholinergic antagonists were bath applied at a flow rate of approximately 4–6 ml/minute: these were the muscarinic antagonist atropine (5 µM, Sigma, Gillingham, UK, https://www.sigmaaldrich.com/united-kingdom.html), the nonselective nAChR antagonist, mecamylamine (MCA; 50 µM), the selective α7*nAChR antagonist at low nanomolar concentrations, methyllycaconitine (MLA; 20 nM), the non‐α7*nAChR antagonist at low micromolar concentrations, dihydro‐β‐erythroidinne (DHβE; 1 µM), and the specific α7*nAChR positive allosteric modulator, PNU 120596 (10 µM; AbCam Biochemicals, Cambridge UK, http://www.abcam.com/). In some experiments, the following drugs were added to the aCSF: tetrodotoxin (TTX; 1 µM; Sigma) to block voltage‐gated Na^+^ channels, D‐(‐)−2‐amino‐5‐phosphonopentanoic acid (D‐AP5; 50 µM) to block NMDA receptors, and 2,3‐dioxo‐6‐nitro‐1,2,3,4‐tetrahydrobenzo[f]quinoxaline‐7‐sulfonamide (NBQX; 20 µM) to block AMPA receptors. Unless stated, drugs were obtained from Tocris Bristol, UK, http://www.tocris.com/.

### Organotypic Spinal Cord Culture

C57/Bl6 mice (9–14 days) were anaesthetized with sodium pentobarbitone (60 mg/kg; intraperitoneal [I.P.]), perfused transcardially with sucrose aCSF and the thoracolumbar spinal cord was removed and transverse sections cut at 300 µm using a McIlwain tissue chopper. After 48 hours in culture, 1 µM EdU (Invitrogen, Paisley, UK) was added into the medium. PNU 120596 (1 µM), MLA (20 nM) alone or a combination of the two was added to the medium of some slices, while some slices from the same animal were untreated (controls) and the slices were maintained in culture for a further 120 hours.

### In Vivo Application of Cholinergic Drugs

Mice (age 6–8 weeks) were injected intraperitoneally with EdU (0.1 ml at 10 mM) and either PNU 120596 (0.1 ml; 10 mM, number of animals = 7) or saline (0.1 ml, number of animals = 7) daily for a period of 4 days.

### Preparation of Tissue for EdU Detection and Immunohistochemistry

Rats (250 g) or mice used above were anaesthetized with sodium pentobarbitone (60 mg/kg) I.P. and perfused transcardially with 4% paraformaldehyde (PFA). Thoracic (T4–8) and lumbar (L1–4) spinal cord sections of 50 µm were cut on a vibrating microtome. For cultured tissue, slices were fixed in 4% PFA for 4–7 hours at 4°C.

### EdU Localization

For EdU detection in cultures, slices (protected from light) were incubated in 320 µl distilled water, 25 µl 2 M Tris buffer, 50 µl 10 mM copper sulphate, 5 µl Azide^594^, and 100 µl 0.5 M ascorbic acid for 30 minutes, then washed in Tris buffer (2 × 10 min). For EdU detection in sections, the same protocol was followed except 5 µl biotinylated azide (1 mM) was used instead of Azide^594^. This was detected by incubating sections in Streptavidin Alexa^555^ (1:1,000 in PBS + 0.1% Triton, Invitrogen) for 1 hour.

### Immunohistochemistry

Immunofluorescence was performed with antibodies against choline acetyl transferase (ChAT; goat, 1:500; Millipore, Watford, UK, http://www.merckmillipore.com/GB/), cluster of differentiation 24 (CD‐24; rat, 1:500; BD, Biosciences, Oxford UK, http://www.bdbiosciences.com/eu/home), polycystic kidney disease 2‐like 1 (PKD2L1, 1:1,000; rabbit, Abcam, Biochemicals), PanQKI (mouse, UC Davis/NIH Neuromab Facility, Davis, CA, http://neuromab.ucdavis.edu/, N147/6, 1:2), glial fibrillary acidic protein (GFAP, mouse, UC Davis/NIH Neuromab Facility N206A/8 1:100), α1 Na^+^/K^+^ ATPase (NKAα1;rabbit, Epitomics, Burlingame, CA, http://www.epitomics.com/, 1:1,000), (beta tubulin III (Tuj1, chicken, 1:500 Neuromics, Edina, MN, http://www.neuromics.com/antibodies), Sox2 (goat, 1:1,000 Santa‐Cruz), and NeuN (mouse, Millipore, 1:1,000). Antibodies were detected with appropriate Alexa^488^ conjugated secondary antibodies.

### Image Capture and Manipulation

Sections were imaged using a Zeiss LSM510 Meta laser scanning confocal microscope equipped with argon (λex = 488 nm) and He‐Ne (λex = 543 nm) lasers. Images were captured using Carl Zeiss ZEN software and images adjusted for brightness, contrast, and intensity using CorelDraw16 software. Figures are single plane confocal images.

### Analysis of Data

For electrophysiology, changes in membrane potential were recorded following ACh application and all data expressed as mean ± SE. Drug effects were determined using paired *t*‐tests. When the CSFcCs were separated into different subtypes, one‐way analysis of variance (ANOVA) with post hoc Bonferroni tests were used to determine the effects of ACh and two‐way ANOVAs with post hoc Bonferroni tests to determine effects of cholinergic modulators on the ACh responses (*n* = no. of cells).

EdU‐positive cells and cells also immunopositive for the specific antibodies were counted in the white and grey matter and the CC region (within 10 µm of the abluminal edge of the ependymal cells) directly through visualization down a microscope (×40 magnification). Cells were mapped, counted, and checked by two investigators. Counts are given as mean number of EdU‐positive cells per 300 µm slice in cultured slices or per 50 µm section for in vivo treatment (*n* = no. of slices or sections, *N* = no. of animals). All data are expressed as means ± SE and for statistical analysis, one‐way ANOVAs with post hoc Bonferroni tests determined differences in the numbers of proliferating cells between control and PNU treated slices and percentages of colocalization.

## Results

### Cholinergic Structures Closely Appose Both Ependymal Cells and CSFcCs

**Figure 1:**
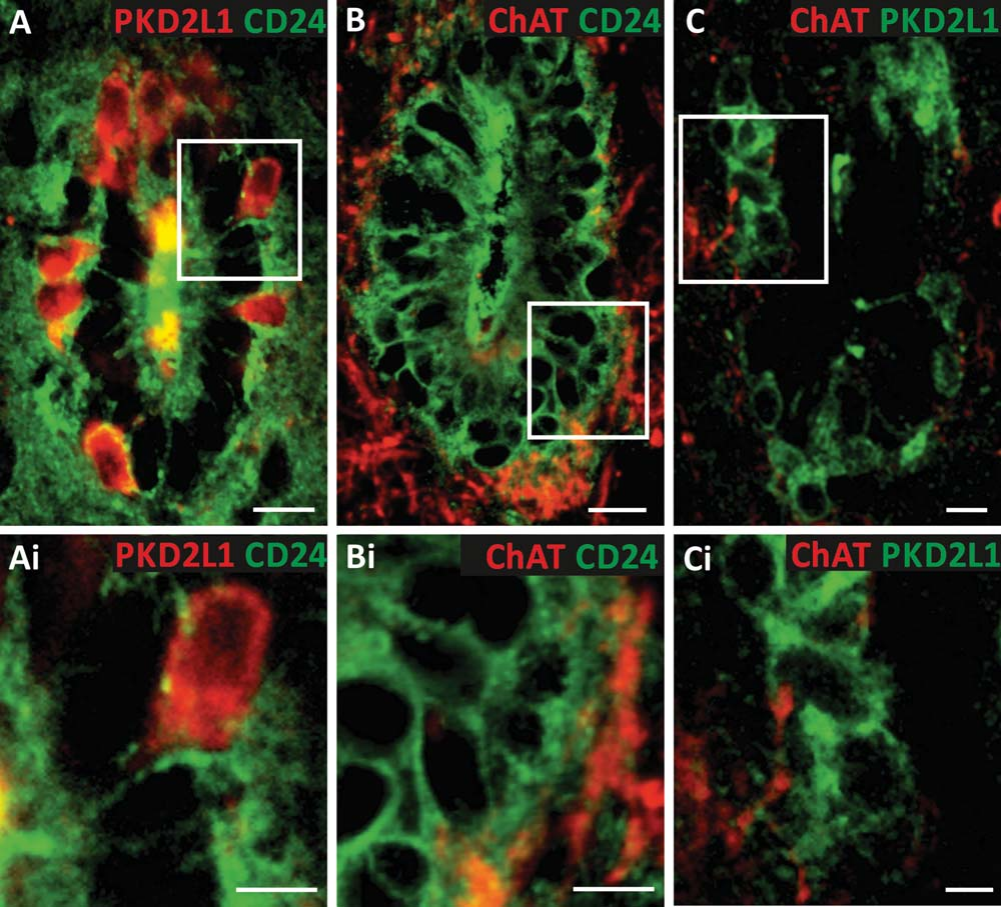
Cholinergic structures are found in close apposition to ependymal cells and cerebrospinal fluid contacting cells (CSFcCs). **(A):** Immunohistochemistry for the CSFcC marker PKD2L‐1 (red) and the ependymal cell marker CD24 (green). **(B):** Combined immunohistochemistry for choline acetyltransferase (ChAT, red) and CD24 (green). **(C):** Combined immunohistochemistry for ChAT (red) and PKD2L1 (green). Scale bars = 10 µm. **(Ai, Bi, Ci):** Higher magnification views of the areas boxed in (A, B, C), respectively. Scale bars = 5 µm.

ChAT immunopositive structures were closely apposed to ependymal cells identified as CD24 +ve 19 (Fig. 1A, 1B) and CSFcCs as PKD2L1 +ve 20 (Fig. 1A, 1C), indicating that cholinergic terminals are ideally located to influence both cell types in this CC region.

### Ependymal Cells and CSFcCs Are Acutely Depolarized by Activation of Cholinergic Receptors

**Figure 2:**
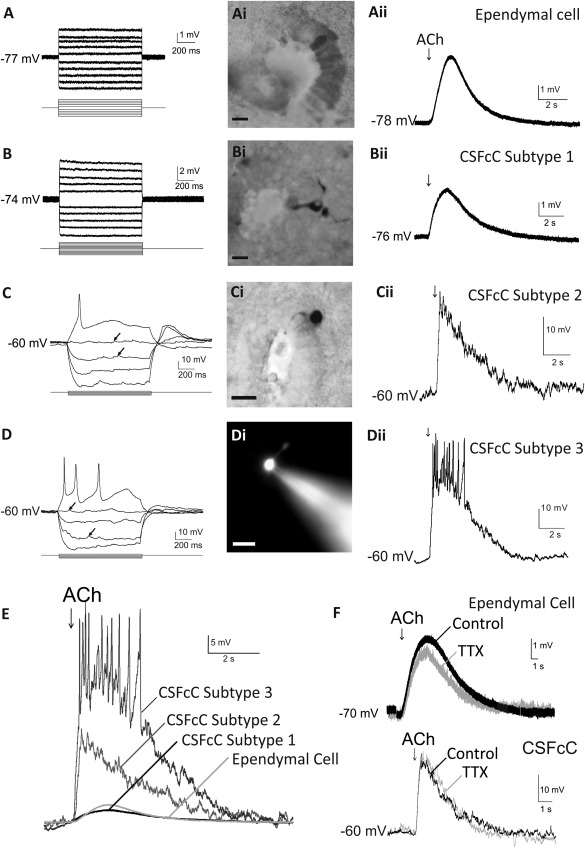
Acetylcholine has profound depolarizing effects on ependymal cells and cerebrospinal fluid contacting cell (CSFcCs). **(A–D):** Responses of ependymal cells and CSFcCs to depolarizing and hyperpolarizing current pulses (A and B, ± 100 pA; C and D, + 10 to −30 pA). Arrows denote synaptic potentials. These responses, together with the morphology of the cells that had been filled with neurobiotin **(Ai‐Ci)** and rhodamine **(Di)**, were used for identification of cell types. Scale bars = 10 µm. **(Aii–Dii):** Responses of cells to pressure application of acetylcholine (ACh); arrows denote puff applied. **(E):** Overlay of the responses to ACh in different cells to show the difference in amplitude of the depolarization. **(F):** Responses to ACh recorded in the presence (tetrodotoxin [TTX]) and absence (control) of TTX. Abbreviations: ACh, acetylcholine; CSFcC, cerebrospinal fluid contacting cell; TTX, tetrodotoxin.

Ependymal cells and CSFcCs were distinguished by their voltage responses to current injections and the morphology revealed by intracellular dye‐loading. Whole cell patch clamp recordings in acute spinal cord slices revealed that ependymal cells had a low input resistance (IR, 96.3 ± 12.6 MΩ, *n* = 79 cells) and no spontaneous activity (Fig. 2A). Ependymal cells also exhibited gap junction coupling between cells, illustrated by transfer of neurobiotin into neighbouring cells, as expected from previous studies 18. Dye filling confirmed ependymal cells by a lack of process into the CC (Fig. 2Ai). CSFcCs were categorized into three subtypes: subtype 1 cells were defined by passive responses to current injections, no spontaneous activity and a small IR (66 ± 9 MΩ; *n* = 14 cells; Fig. 2B). Subtype 2 cells produced an action potential at the beginning of the positive current injection, exhibited spontaneous activity in the form of synaptic potentials that could be blocked by TTX (not shown) as seen previously 7 and had a large IR (2668 ± 130 MΩ; *n* = 130 cells; Fig. 2C). Subtype 3 cells could produce multiple action potentials in response to positive current pulses (Fig. 2D). These cells also exhibited spontaneous activity and an average IR (2336 ± 149 MΩ; *n* = 52 cells) that was not significantly different from that of subtype 2 CSFcCs. Cells were confirmed as CSFcCs post‐recording by the presence of a process into the CC (Fig. 2Bi–2Di).

Pressure ejection of ACh (3 mM) depolarized 100% of CSFcCs (*n* = 82 cells) and ependymal cells (*n* = 36 cells; Fig. 2Aii–2Dii). The responses of each cell type to ACh varied in amplitude for ependymal cells (3.0 ± 0.3 mV; *n* = 36), subtype 1 CSFcCs (2.2 ± 0.2 mV; *n* = 17 cells), subtype 2 CSFcCs (18.9  ± 1.2 mV; *n* = 46 cells), and subtype 3 CSFcCs (26.2 ± 2.2 mV; *n* = 19 cells). A one‐way ANOVA revealed significant differences in the amplitude of the cholinergic response of ependymal cells, subtype 1 CSFcCs, subtype 2 CSFcCs, and subtype 3 CSFcCs (*p* < 0.001). Post hoc tests revealed no significant difference between the amplitude of the cholinergic response in ependymal cells and subtype 1 CSFcCs (*p* = 1); however, the amplitudes of these responses were significantly smaller than those of both subtype 2 and subtype 3 CSFcCs (*p* < 0.001; Fig. 2E). The response to ACh was likely to be a direct effect on ependymal cells and CSFcCs as the responses were not significantly decreased in the presence of TTX, AP5, and NBQX (*p* < 0.05; *n* = 3–5 cells; Fig. 2F).

### Cholinergic Responses of Ependymal Cells and CSFcCs Are Mediated by nAChRs

**Figure 3:**
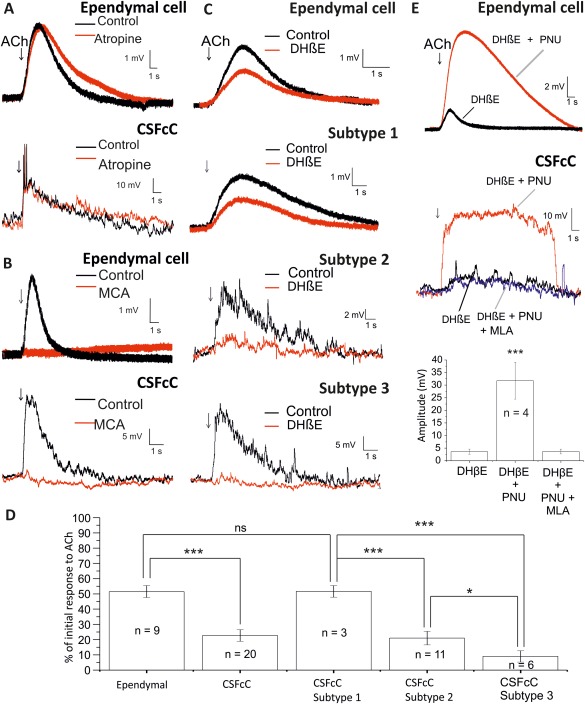
Both α7*nAChRs and non‐α7*nAChRs contribute to the depolarizations. **(A):** Acetylcholine (Ach) responses of cerebrospinal fluid contacting cells (CSFcCs) or ependymal cells in the presence and absence (labeled control) of the muscarinic receptor antagonist, atropine (5 µM). **(B):** ACh responses of CSFcCs or ependymal cells in the presence and absence (labeled control) of the nonselective nicotinic receptor antagonist, mecamylamine (50 µM). **(C):** ACh responses of ependymal cells and CSFcC subtypes in the presence and absence of dihydro‐β‐erythroidinne (DHβE) (1 µM) **(D):** Mean amplitude ± SE of the response to ACh during the bath application of DHβE expressed as a percentage of the initial response to ACh, for ependymal cells, CSFcCs and CSFcC subtypes. *, *p* < 0.05; **, *p* < 0.01; ***, *p* < 0.001. **(E):** ACh responses of ependymal cells and CSFcCs in the presence of DHβE (1 µM) alone or with DHβE and PNU 120596 (10 µM). In the bottom trace, the ACh responses were also tested in the presence of DHβE, PNU 120596, and α7*nAChR antagonist methyllycaconitine (MLA) (20 nM). The bar chart shows the mean data for the effects of PNU 120596 in the presence of DHβE and MLA. Abbreviations: ACh, acetylcholine; CSFcC, cerebrospinal fluid contacting cell; DHβE, dihydro‐β‐erythroidinne; MCA, mecamylamine; MLA, methyllycaconitine.

Atropine (5 µM), the muscarinic selective antagonist, had no significant effect on ACh responses in either cell type (regardless of CSFcC subtype; Fig. 3A). The nicotinic‐selective antagonist, MCA (50 µM), significantly reduced the ACh response in both ependymal cells (2.9 ± 0.9 mV to 0.2 ± 0.1 mV; *n* = 6 cells; *p* < 0.05) and all CSFcCs, regardless of subtype (17.9 ± 2.3 mV to 1.1 ± 0.5 mV; *p* < 0.001; *n* = 6 cells; Fig. 3B).

DHβE, a selective non‐α7*nAChR antagonist at the concentration used (1 μM), decreased the response of both ependymal cells (3.7 ± 0.7 mV to 1.9 ± 0.4 mV; *n* = 9 cells) and CSFcCs (16.1 ± 1.8 mV to 3.0 ± 0.5 mV; *n* = 20 cells, Fig. 3C). Separating the CSFcCs into the defined subtypes revealed that there was a differential effect of DHβE on the specific subtypes. The ACh response (expressed as a percentage of the control response) in the presence of DHβE was 52% ± 4% (*n* = 3 cells) for subtype 1, 22% ± 3% (*n* = 11 cells) for subtype 2 and 9% ± 4% (*n* = 6 cells) for subtype 3. This degree of antagonism by DHβE was significantly different for each subtype (*p* < 0.05, Fig. 3D).

The contribution of α7*nAChRs was investigated using MLA, which is a selective α7*nAChR antagonist at the concentration used (20 nM, 21). Bath application of MLA significantly decreased the amplitude of the cholinergic response in ependymal cells from 4.1 ± 0.8 mV to 3.5 ± 0.8 mV (*n* = 6 cells; *p* < 0.05). The amplitude of the cholinergic response in CSFcCs was also significantly decreased in MLA from 24.2 ± 3.6 mV to 19.7 ± 3.4 mV (*n* = 8 cells; *p* < 0.05).

All ACh responses remaining in the presence of DHβE could also be profoundly potentiated by the selective α7*nAChR modulator, PNU 120596 (10 μM), regardless of cell type. A significant increase in the amplitude of the cholinergic response of ependymal cells (1.5 ± 0.2 mV to 7.3 ± 1.9 mV; *n* = 4 cells; *p* < 0.05) and all CSFcCs, regardless of subtype (3.6 ± 0.7 mV to 27.6 ± 4.0 mV; *n* = 10 cells; *p* < 0.001) was observed in the presence of PNU 120596 (Fig. 3E). This potentiation could be completely antagonized by MLA, with responses in DHβE, PNU 120596, and MLA not significantly different from those observed in the presence of DHβE alone (82% ± 20%; *n* = 4 cells; *p* = 0.39, Fig. 3E).

### Activation of α7*nAChRs Increases Proliferation of Ependymal Cells in Cultured Spinal Cord Slices

**Figure 4:**
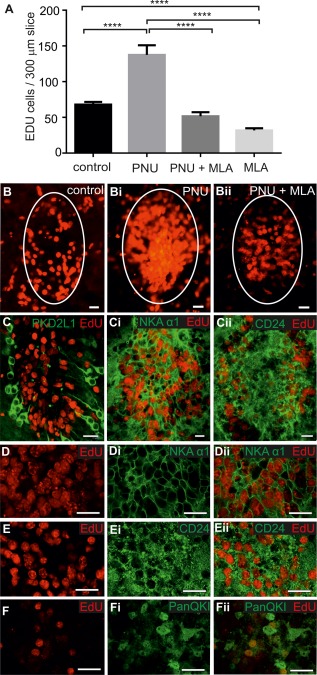
Administration of the selective α7*nAChR modulator, PNU 120596 increased proliferation of ependymal cells in spinal cord cultures. **(A):** The numbers of 5‐ethynyl‐2'‐deoxyuridine (EdU)‐positive cells per 300 µm slice in the presence of saline alone, PNU 120596 (1 µM) alone, PNU 120596, and the α7*nAChR antagonist methyllycaconitine (MLA) (20 nM) or MLA (20 nM) alone. **(B):** Confocal images showing examples of EdU‐labeled cells in animals treated with saline, PNU 120596 **(Bi)** or PNU 120596 and MLA **(Bii)**. **(C–Cii):** confocal images showing EdU‐labeled cells in the region of the central canal do not contain immunoreactivity for the cerebrospinal fluid contacting cell marker PKD2L1 (C) yet are colocalized with immunoreactivity for the ependymal cell markers NKA α1 **(Ci)** and CD24 (Cii) following treatment with PNU 120596. **(D–Eii):** Higher power confocal images showing EdU‐labeled cells (D, E) are also immunoreactive for NKA α1 **(Di)** and CD24 **(Ei)** as shown by the merged images (Dii, Eii) following treatment with PNU 120596. **(F):** EdU‐labeled cells (F) colocalize with PanQKI immunoreactivity **(Fi, Fii)**. Scale bars = 20 µm. Abbreviations: EdU, 5‐ethynyl‐2'‐deoxyuridine; MLA, methyllycaconitine.

The possibility that α7*nAChR modulation could influence cell proliferation in the spinal cord was tested by the addition of PNU 120596 (1 µM) to the culture medium in organotypic spinal cord slice cultures. The numbers of EdU‐positive cells around the CC in PNU 120596 treated slices were significantly higher (137.5 ± 13.5; *n* = 13 slices, *N* = 6 animals) than those in saline treated control slices (76.2 ± 4.9; *n* = 16 slices, *N* = 6 animals; *p* < 0.001); (Fig. 4A, 4B). Addition of PNU 120596 in the presence of the α7*nAChR antagonist MLA (20 nM) significantly reduced the number of EdU‐positive cells (51.6 ± 5.6; *n* = 14 slices, *N* = 6 animals; *p* < 0.0001) compared with those observed in the presence of PNU 120596 alone. Indeed, the number of EdU‐positive cells in PNU 120596 and MLA was not significantly different to those observed in the saline control. To test whether α7*nAChRs were being endogenously activated in the spinal cord cultured slice, MLA alone was applied, which significantly reduced cell proliferation compared with control (Fig. 4A). This indicates that activation of these cholinergic receptors contributes to endogenous levels of proliferation.

### Activation of α7*nAChRs Increases Proliferation of Ependymal Cells In Vivo

**Figure 5:**
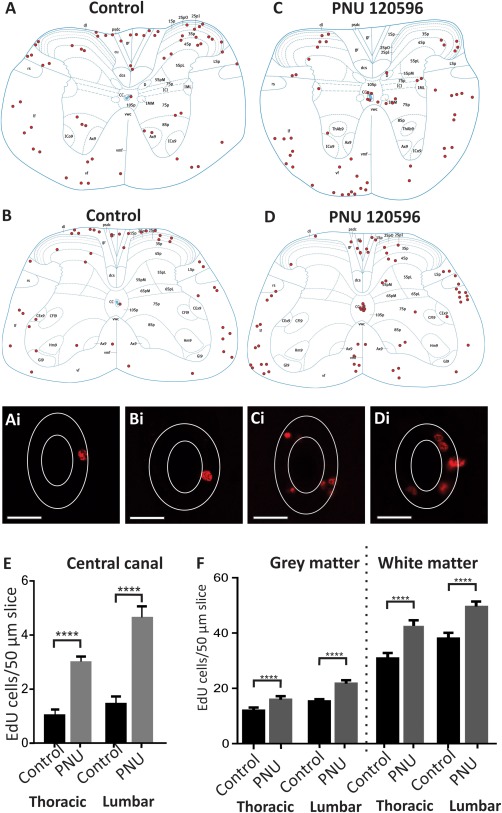
In vivo administration of the cholinergic modulator PNU 120596 increased the number of 5‐ethynyl‐2'‐deoxyuridine (EdU)‐labeled cells in both thoracic and lumbar regions. **(A–D):** Representative diagrams showing the distribution of EdU‐labeled cells in control sections in thoracic (A) and lumbar (B) regions compared with PNU 120596 treated sections at thoracic (C) and lumbar (D) levels. **(Ai–Di):** Confocal images of the EdU‐labeled cells surrounding the central canal (CC) from the representative diagrams above. Scale bars = 20 µm. **(E, F):** Average number of EdU‐labeled cells per 50 µm section ± SE in the area of the CC (E), grey and white matter (F) of thoracic and lumbar spinal cord sections in control and PNU 120596 treated animals. ****, *p* < 0.0001. Abbreviation: EdU, 5‐ethynyl‐2'‐deoxyuridine.

To determine whether the α7*nAChR‐mediated enhancement of proliferation in slice culture could be extended to the whole animal and could also be observed in adults, the effects of in vivo application of the selective α7*nAChR modulator PNU 120596 on cell proliferation in the CC, grey, and white matter of the spinal cord were tested. Sections were analyzed from thoracic and lumbar spinal cord regions and the locations of EdU‐positive cells were mapped (Fig. 5).

In the CC area (Fig. 5E), the average numbers of EdU‐positive cells per section in control animals (*N* = 4 animals) injected with saline were 1.0 ± 0.2, (*n* = 35 sections) in the thoracic region and 1.4 ± 0.3 (*n* = 40 sections) in the lumbar region. In comparison with control sections, injection of PNU 120596 significantly increased (*p* < 0.0001) the average number of EdU‐labeled cells per section in the CC area in both the thoracic (3.0 ± 0.2, *n* = 40 sections, *N* = 4 animals) and lumbar (4.6 ± 0.4, *n* = 40 sections) regions.

In the grey matter (Fig. 5F), not including the CC region already counted, the average numbers of EdU‐labeled cells per section in control animals were 11.9 ± 1.2 (*n* = 35 sections) in thoracic sections and 15.2 ± 0.8 (*n* = 40 sections) in lumbar sections. Injection of PNU 120596 caused a significant (*p* < 0.0001) increase in the number of EdU‐labeled cells in the grey matter of both the thoracic (15.9 ± 1.3 cells per section, *n* = 40 sections) and lumbar (21.8 ± 1.3 cells per section, *n* = 40 sections) spinal cord compared with control. In the white matter, (Fig. 5F) control animals had EdU‐labeled cells in both the thoracic (30.6 ± 2.0 cells per section, *n* = 35 sections) and lumbar (37.8 ± 2.7 cell per section, *n* = 40 sections) regions. Once again, injection of PNU 120596 caused a significant (*p* < 0.0001) increase in EdU‐labeled cells in the white matter of thoracic (42.3 ± 2.0 cells per section, *n* = 40 sections) and lumbar (49.5 ± 1.9 cells per section, *n* = 40 sections) regions.

### Identity of Proliferating Cells in the CC

**Figure 6:**
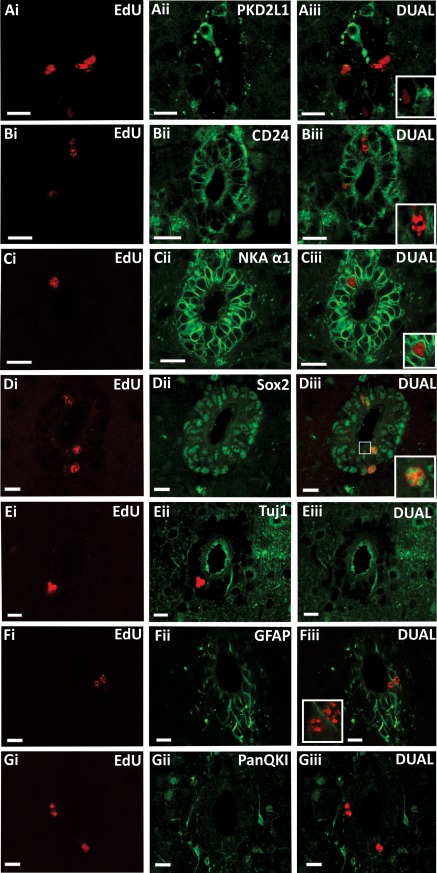
Following in vivo PNU 120596 administration, 5‐ethynyl‐2'‐deoxyuridine (EdU)‐labeled cells surrounding the central canal (CC) are proliferating, yet undifferentiated. **(A–D):** Confocal images following PNU 120596 application in vivo showing EdU‐labeled cells surrounding the CC **(Ai)** are distinct from the population of PKD2L1 immunoreactive cerebrospinal fluid contacting cells **(Aii)** as seen in the merged image **(Aiii).** EdU‐labeled cells **(Bi, Ci)** are colocalized with immunoreactivity for the ependymal cell markers CD24 **(Bii)** and NKA α1 **(Cii)** as shown in the merged images **(Biii, Ciii)**. EdU‐labeled cells contain immunoreactivity for the transcription factor Sox2 (EdU **[Di]**, Sox2 [**Dii]**, merged image [**Diii]**; boxed area shows a higher magnification of a dual‐labeled cell). EdU‐labeled cells show no colabeling with the neuronal marker Tuj1 **(Ei–Eiii)**, the astrocyte marker glial fibrillary acidic protein **(Fi–Fiii)** or the oligodendrocyte marker PanQKI **(Gi–Giii)**. Scale bars = 10 µm. Abbreviations: EdU, 5‐ethynyl‐2'‐deoxyuridine; GFAP, glial fibrillary acidic protein; NKAα1, Na^+^/K^+^ ATPase α1.

As both CSFcCs and ependymal cells were shown to be acutely depolarized by activation of α7*nAChRs, we next established which type of cells were proliferating in both cultured slices and in vivo. Using antibodies for CD24 or NKAα1 22 as markers for ependymal cells and PKD2L1 as a marker of CSFcCs, dual labeling for EdU was carried out on treated slices. Colocalization of anti‐PKD2L1 and EdU labeling was not observed in either condition (*n* = 13 slices, *N* = 6 animals for cultured slices; Fig. 4C and *n* = 15 sections, *N* = 5 animals for in vivo treatment; Fig. 6A); however, colocalization of anti‐CD24 or NKAα1 and EdU labeling was always seen in both cultured slices and after in vivo treatment (*n* = 13 slices, *N* = 6 animals for cultured slices; Fig. 4Ci–4E and *n* = 16 sections, *N* = 5 animals for in vivo treatment; Fig. 6B, 6C). Furthermore, EdU in the ependymal layer always colocalized with Sox2, a transcription factor associated with ependymal cells 23, in both the in vivo experiments (Fig. 6D) and cultured spinal cord slices. Taken together, the presence of Sox2 in the nucleus and CD24 and NKAα1 in the membranes surrounding all EdU‐positive cells, is consistent with activation of α7*nAChRs selectively initiating proliferation in ependymal cells and not CSFcCs.

The identity of those cells in the CC that had proliferated in vivo was further tested using dual labeling techniques after in vivo treatment with PNU 120596. EdU‐labeled cells surrounding the CC contained immunoreactivity for the transcription factor Sox2, consistent with a stem cell identity (24; Fig. 6D). Furthermore, EdU‐labeled cells did not contain immunoreactivity for class III ß tubulin (Tuj1) a marker for immature and mature neurons (25; Fig. 6E), the reactive glial cell marker GFAP (26; Fig. 6F) or the oligodendrocyte marker PanQKI (27, 28; Fig. 6G). There were never any neuronal nuclei marker (NeuN) immunopositive neurons (labeling mature neurons) in the vicinity of the CC (not shown). The colocalization of EdU with Sox2, CD24, and NKAα1 normally expressed by ependymal cells, but not GFAP nor PKD2L1 which are expressed by other ependymal layer cell types, is consistent with the EdU positive ependymal layer cells being ependymal cells with stem cell characteristics.

As the newly proliferated cells in the CC region of the intact spinal cord seem to be maintained in a state of readiness as stem cells, we hypothesized that signals to induce differentiation were absent and the α7nAChR activation could be strongly promoting proliferation over differentiation. As preparation of the spinal cord cultures necessarily involves damage to the system again, we hypothesized this may be sufficient to trigger such differentiation. In control conditions, cells in the CC were neither NeuN nor Tuj1 immunoreactive, while the proportions of EdU‐labeled cells that were also labeled for GFAP (2.4 ± 1.2 %) or PanQKI (7.3 ± 2.4 %) were quite low. PNU 120596 application resulted not only in an increase in proliferation but also significantly (*p* = 0.02) increased the proportion of cells that were colabeled for PanQKI to 15.1 ± 2.6 % (Fig. 4F). There were still no cells colabeled for EdU and either NeuN or Tuj1 nor was there a significant change in the level of colabeling for EdU and GFAP (4.0 ± 1.0 %). This suggests that presence of an α7nAChR potentiator results in the proliferating cells preferentially differentiating along an oligodendrocyte lineage.

### Identity of Proliferating Cells in the Other Spinal Cord Regions

**Figure 7:**
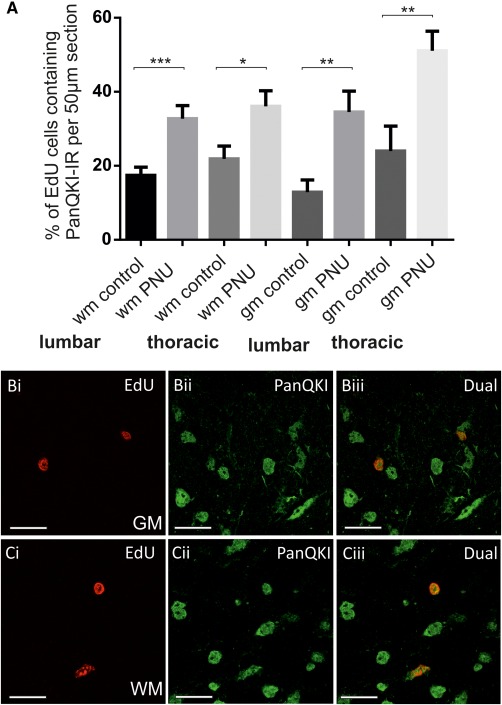
Following in vivo PNU 120596 administration, 5‐ethynyl‐2'‐deoxyuridine (EdU)‐labeled cells in the white and grey matter preferentially become oligodendrocytes. **(A)** Average percentage ± SE of 5‐ethynyl‐2'‐deoxyuridine (EdU) and PanQKI colabeled cells per 50 µm section in the grey matter (gm) and white matter (wm) of thoracic and lumbar spinal cord sections in control and PNU 120596‐treated animals. *, *p* < 0.05; **, *p* < 0.01; ***, *p* < 0.001. **(B, C):** Confocal images showing examples of EdU and PanQKI‐IR colocalization in grey **(Bi–Biii)** and white **(Ci–Ciii)** matter. Scale bars = 20 µm. Abbreviations: EdU, 5‐ethynyl‐2'‐deoxyuridine; PNU, PNU 120596; WM, white matter.

In the grey and white matter of the spinal cord, the numbers of cells colocalized for EdU and either NeuN or GFAP were negligible. These were not changed by the application of PNU 120596. However, there were moderately high percentages of EdU immunoreactive cells that were also colabeled for PanQKI in thoracic (grey matter 24.0% ± 6.7%, white matter 21.8% ± 3.4%) and lumbar (grey matter 12.9% ± 3.3%, white matter 17.4% ± 2.2%) sections. These proportions were significantly (*p* = 0.0001) increased by PNU 120596 in both thoracic (grey matter 51.1% ± 5.3%, white matter 36.1% ± 4.2%) and lumbar (grey matter 34.6% ± 5.7%, white matter 32.8% ± 3.6%) sections (Fig. 7). This indicates that the newly proliferated cells in the intact white and grey matter of the spinal cord also preferentially express oligodendrocyte markers.

## Discussion

This study provides the first evidence that cell proliferation in the neurogenic niche of the postnatal mammalian spinal cord is significantly enhanced by the potentiation of endogenous ACh through activation of α7*nAChRs in both organotypic slice cultures and in vivo. Although both ependymal cells and CSFcCs of the mammalian spinal cord responded robustly to ACh in acute spinal cord slices, our data revealed it was only the ependymal cells that were capable of cholinergic receptor induced proliferation. In addition, α7*nAChR stimulation resulted in proliferation of cells in both the white and grey matter. There was a significant increase in the proportion of newly proliferated cells that expressed oligodendrocyte markers.

### Cholinergic Responses of Ependymal Cells and CSFcCs

The combination of immunohistochemistry demonstrating the presence of cholinergic structures closely apposing ependymal cells and CSFcCs and electrophysiology demonstrating maintenance of the ACh response in TTX, suggests that the ACh response is mediated by receptors located directly on the membrane of both cell types. The cholinergic structures may be either synaptic terminals or dendrites; in other central nervous system (CNS) regions, both dendrites and axon terminals have the capacity to release acetylcholine 29. There could be an alternative source of ACh or choline within the CSF 30, which both cell types contact. The variation in the size of the ACh response in different CSFcC subtypes is likely to correspond to a difference in receptor density or proportions of receptors mediating the response.

Both antagonisms of the cholinergic response by DHβE and potentiation of the response by PNU 120596 are in agreement with in situ hybridization studies, which reveal the expression of α7*nAChR 31 and non‐α7*nAChR subunits including α2–4 and β2 32. In addition, an α7 nAChR subunit reporter mouse made by the Gensat project (Gensat 2012) showed some GFP expressing CSFcCs. As DHβE is a preferential antagonist at α4*nAChRs and β2*nAChRs 33, 34, it is likely that α4β2*nAChRs could be contributing to the cholinergic response in both ependymal cells and CSFcCs, however, this needs further confirmation. The indication of the presence of α7*nAChRs was further supported by the antagonism of PNU 120596 potentiation by MLA in both acute and cultured slices.

### Functional Implications of Cholinergic Modulation of Cells in the Neurogenic Niche

The importance of this cholinergic influence on the CC neurogenic niche is maintained into adulthood since it was observed in both cultured slices obtained from juvenile animals and fully mature mice, using the in vivo preparation. The higher number of proliferating cells in the control cultured spinal cord slices compared with the in vivo situation is likely due to the fact that slice culture is more representative of an injury state in which increased proliferation is observed 4, 5. This further indicates that cholinergic enhancement of proliferation can occur in physiological and pathological situations. Indeed, the reduction of proliferation by application of only α7*nAChR antagonists in culture suggests that these receptors may be necessary and sufficient for injury induced proliferation to occur.

There are a number of functional implications to consider with regards to the effects of the cholinergic modulators on ependymal cells and CSFcCs. The fact that PNU 120596 has an effect in cultured slices suggests that the source of ACh is local and maintained in this reduced preparation. An initial consideration is whether the two possible sources of ACh, axodendritic, or CSF, are mediating different functional effects. For example, structures containing ACh closely apposing both cell types could be important for ensuring that newly generated cells are integrated within the spinal cord circuitry, responding to neighboring cells and contributing to specific pathways. This idea is similar to that recently proposed in the subventricular zone where newly identified cholinergic neurons are sufficient to control neurogenic proliferation 15. In the case of CSFcCs, it has previously been hypothesized that these cells act as sensory neurons, sensing the composition of the CSF 35, and this is supported as they can respond to changes in pH and ATP 7. This study, however, provides evidence that they are far more complex than simple CSF sensors; they could be modulated by other neurons. The origin of the cholinergic structures influencing either ependymal cells or CSFcCs is currently unknown, although it could be speculated that they are from partition or CC cluster cholinergic interneurons located in lamina X 36. One possible source is a small cluster of cholinergic interneurons in the CC region that provide c boutons onto motoneurons 16; their close proximity to the ependymal cell layer would enable release from either dendrites or axon terminals. Given the central location of ependymal cells and CSFcCs within the spinal cord, they are well placed to receive inputs regarding sensory, autonomic and motor functions. Alternatively or indeed additionally, ACh or choline within the CSF 37 could act in a paracrine manner coordinating a widespread effect on populations of ependymal cells and CSFcCs throughout the spinal cord. This is less likely due to the fact that MLA reduced proliferation in the culture, where CSF is washed out.

Both sources of ACh could mediate effects on the proliferation, maturation, and survival of both cell types. With regard to CSFcCs, our study did not reveal α7*nAChR mediated proliferation of CSFcCs, although these cells were highly responsive to activation of these receptors. It is also possible that cholinergic receptor activation of CSFcCs or indeed other intermediary cells could release factors which in turn contribute to the ependymal cell proliferation. However, our evidence from the recordings in acute spinal cord slices suggests that proliferation of ependymal cells could be due to a direct activation of cholinergic receptors on these cells. Cholinergic stimulation of ependymal cells is likely to induce an increase in intracellular Ca^2+^, as observed in other non‐neuronal CNS cells 38, 39, leading to a number of downstream effects. Interestingly, following increases in intracellular Ca^2+^, the coupled nature of ependymal cells would enable propagation of Ca^2+^ waves as observed in radial glial cells during embryonic neurogenesis and neural progenitor cells in the postnatal subventricular zone 40, 41. Ependymal cells of the spinal cord proliferate to maintain the ependymal cell population under normal conditions 2. This study demonstrates that cholinergic modulation enhances this proliferation and as this system is readily accessed pharmacologically, this provides an avenue for therapeutic intervention.

### Implications for New Avenues in Spinal Cord Regeneration

An important finding in the CC was the strong colocalization of EdU with the transcription factor Sox2. Sox2 is considered a marker of neural stem cells since it inhibits neuronal differentiation and maintains progenitor characteristics 24, which is also evident in the spinal cord ependymal cell population 42. Thus, enhancing the actions of α7*nAChRs leads to increased production of neural stem cells. We did not find evidence of differentiation of the newly proliferated cells in the intact CC region of spinal cord since EdU was not colocalized with markers of neurons or glial cells, but our short recovery period is likely to have precluded this. Alternatively, signals for appropriate differentiation may have been absent in the CC of spinal cords. However, in the cultured spinal cord slices, we found that a higher proportion of the EdU positive CC cells was also colabeled with anti‐PanQKI in PNU 120596 treated animals compared with control. This suggests that in this spinal cord slice culture, differentiation along the oligodendrocyte lineage is more prominent when α7*nAChRs are activated. In fact, it is known that mammalian spinal ependymal cells can differentiate in some conditions, indeed they are the only spinal cells capable of generating progeny of multiple fates 5. In a mouse model of multiple sclerosis, ependymal cells not only proliferated but also migrated and differentiated into neurons 6. A recent study in zebrafish was the first to demonstrate that a neurotransmitter (dopamine) could promote spinal motor neuron generation in the lesioned spinal cord 9. Therefore, similar to other CNS regions 43, identification of appropriate signals to direct differentiation and improve survival and integration of endogenously produced neural precursors will be amongst the next steps towards spinal repair.

Another important finding was the increased number of EdU‐labeled cells in the white and grey matter of the spinal cord following PNU 120596 administration. These cells also expressed Sox2, indicating the potential to increase stem cell production outside of the CC. Indeed, PNU treatment not only increased EdU labeling, but resulted in an increased proportion of EdU‐labeled cells being immunoreactive for PanQKI. While the identity of the cells contributing to the α7*nAChR induced proliferation in the white and grey matter remains to be determined, one potential source could be the meninges 44. It is clear though that the α7*nAChR could be a viable target to increase the numbers of oligodendrocytes in the spinal cord, perhaps following injury or in demyelinating diseases such as multiple sclerosis.

## Summary

In conclusion, this study is the first to demonstrate that neurotransmitter mediated manipulation can enhance spinal cord cell proliferation in mammals. One likely source of these cells is the ependymal cell layer 4, 5, consistent with our observations here. The factors determining the fates of these cells require further elucidation, but our data provide new potential for future therapeutic interventions since the cholinergic system can be manipulated by drugs that cross the blood brain barrier.

## Author Contributions

L.C.: design of some experiments, collection and/or assembly of data, data analysis and interpretation, manuscript writing; L.A. and J.D.: collection and/or assembly of data, data analysis and interpretation, manuscript writing; I.J.E. and L.N.: collection and/or assembly of data, data analysis and interpretation; J.D. and S.D.: conception and design, financial support, data analysis and interpretation, manuscript writing, final approval of manuscript.

## Disclosure of Potential Conflicts of Interest

The authors indicate no potential conflicts of interest.

## Supporting information

Supplementary Information
