# Supplementary material for: Cholinergic Enhancement of Cell Proliferation in the Postnatal Neurogenic Niche of the Mammalian Spinal Cord
Source: Stem Cells. 2015 Jun 26;33(9):2864–76. doi: 10.1002/stem.2077 (PMC4737096; doi:10.1002/stem.2077)
Supplement: Supplementary file 1 — Supplementary Information [file STEM-33-2864-s001.docx]

**Methods and Materials**

*Animals*

Wistar rats (P9-adult) or C57/Bl6 mice (P9-adult) of either sex were used in line with the UK Animals (Scientific Procedures) Act 1986 and ethical standards set out by the University of Leeds Ethical Review Committee. Every effort was made to minimise the number of animals used and their suffering.

*Immunohistochemistry*

Rats (250 g) or mice (6-8 weeks) were anaesthetised with sodium pentobarbitone (60 mg/kg) I.P. and perfused transcardially with 4% paraformaldehyde (PFA). Spinal cords were dissected and post-fixed in 4% PFA overnight, washed in 0.1 M phosphate buffer (PB) and cut (50 µm) from levels T4-8 and L1-4. Sections were incubated in primary antibodies overnight in 0.1 M PBS containing 0.3% triton X-100 and 3% donkey serum. Primary antibodies were goat anti-choline acetyl transferase (ChAT; 1:500; Millipore), rat anti-cluster of differentiation 24 (CD-24; 1:500; BD Pharmingen) and rabbit anti-PKD2L1 (1:1000; Abcam). For CD-24, antigen retrieval was performed before incubation in primary antibody by incubation at 80°C in 10 mM sodium citrate (30 minutes) then washed (3 x 10 minute) in 0.1 M PBS. Sections were incubated in secondary antibodies (2 hours): donkey anti-goat IgG Alexa Fluor ^555^ (1:1000; Invitrogen), donkey anti-rabbit IgG Alexa Fluor ^555^, donkey anti-rabbit Alexa^488^ (1:1000, Invitrogen) or biotinylated donkey anti-rat (1:250; Jackson Immunoresearch) followed by streptavidin-Alexa^488^ (1:1000; Invitrogen). Sections were washed in 0.1 M PBS 3 x 10 minutes, air dried onto glass slides and mounted in Vectashield (Vector Laboratories).

*Slice preparation*

Animals (9-28 days) were anaesthetised with sodium pentobarbitone (60 mg/kg) I.P and perfused transcardially with ice-cold sucrose artificial CSF (aCSF) of the following composition (mM): Sucrose (217), NaHCO_3_ (26), KCl (3), MgSO_4_.7H_2_O (2), NaH_2_PO_4_ (2.5), Glucose (10), CaCl_2_ (1), equilibrated with 95 % O_2_ and 5 % CO_2_. The spinal cord was removed and the lower thoracic spinal cord embedded in agar before cutting 300 µm thick transverse slices using a vibrating microtome (Leica VT1200s, Leica Microsystems, UK). Slices were transferred to a holding chamber containing aCSF (95 % O_2_: 5 % CO_2_; mM): NaCl (124), NaHCO_3_ (26), KCl (3), MgSO_4_.7H_2_O (2), NaH_2_PO_4_ (2.5), Glucose (10), CaCl_2_ (2) and equilibrated at room temperature for 30-60 minutes.

*Whole cell patch clamp electrophysiology*

Whole cell current clamp recordings were made at room temperature from CSFcCs and ependymal cells using an Axopatch-1D amplifier and microelectrodes filled with intracellular solution (mM): K gluconate (110), EGTA (11), MgCl_2_ (2), CaCl_2_ (0.1), HEPES (10), Na_2_ATP (2), NaGTP (0.3). Neurobiotin (0.5 %; Vector Laboratories, USA) and tetramethylrodamine (0.02 %; Invitrogen, USA) were added to visualise the cells post-recording (see [1]). Depolarising and hyperpolarising current pulses of 1 second duration (-50 pA to +50 pA) were applied and input resistance (IR) determined.

ACh (3-10 mM) was pressure ejected locally for 500 ms at a pressure of 10 psi from patch pipettes using a PV800 pneumatic pico pump. Cholinergic antagonists were bath applied at a flow rate of ~4-6 ml/min: these were the muscarinic antagonist atropine (5 µM, Sigma), the non-selective nAChR antagonist, mecamylamine (MCA; 50 µM), the selective α7*nAChR antagonist at low nanomolar concentrations, methyllycaconitine (MLA; 20 nM), the non-α7*nAChR antagonist at low micromolar concentrations, dihydro-β-erythroidinne (DHβE; 1 µM) and the specific α7*nAChR positive allosteric modulator, PNU 120596 (10 µM; AbCam Biochemicals). In some experiments the following drugs were added to the aCSF: tetrodotoxin (TTX; 1 µM; Sigma) to block voltage-gated Na^+^ channels, D-(-)-2-Amino-5-phosphonopentanoic acid (D-AP5; 50 µM) to block NMDA receptors and 2,3-Dioxo-6-nitro-1,2,3,4-tetrahydrobenzo[f]quinoxaline-7-sulfonamide (NBQX; 20 µM) to block AMPA receptors. Unless stated, drugs were obtained from Tocris UK.

### For morphological identification of cells, they were first imaged at the end of recordings using epifluorescence to visualise tetramethylrhodamine. The slice was then fixed in 4 % paraformaldehyde + 0.25 % glutaraldehyde for more detailed post-hoc visualisation. Slices were washed in 0.1 M PB, gelatine embedded and resectioned (50 μm) using a vibrating microtome. Sections were permeabilised in PBS containing 0.1 % triton X-100, incubated in extraavidin-peroxidase (1:250 in PBS) for 36-72 hours, then washed in 0.1 M PBS. Cells were visualised by incubating in diaminobenzadine (0.5 mg/ml in tris buffer) in the presence of 1 % H_2_O_2_ until the cell was visualised.

*Organotypic spinal cord culture*

C57/Bl6 mice (9-14 days) were anaesthetised with sodium pentobarbitone (60 mg/kg; I.P.), perfused transcardially and the thoracolumbar spinal cord was removed and cut at 300 µm using a McIlwain tissue chopper. Lower thoracic spinal cord slices were transferred onto Millicell organotypic filter inserts (Millipore) in a six well plate. Each well contained 1 ml of neurobasal A culture medium with 10 % fetal bovine serum, 1 % L-Glutamine, 1 % penicillin/ streptomycin (all Sigma) at 37 ºC. Plates were maintained in an incubator at 37 ºC and 5 % CO_2_. After 24 hr in culture, the media was replaced with serum-free neurobasal A medium with 2 % B27 supplement, 1 % L-Glutamine, 1 % penicillin/streptomycin. Half the culture medium was then replaced using serum-free medium every two days. After 48 hr in culture 1 µM EdU (5-ethynyl-2-deoxyuridine) was added into the medium. PNU 120596 (1 µM), either alone or in the presence of MLA (20 nM) was added to the medium of some slices, while some slices from the same animal were untreated (controls) and the slices were maintained in culture for a further 120 hours.

The slices were fixed in 4% PFA for 4-7 hr at 4ºC, free floated in 0.1 M PBS before washing in 0.1 M PBS with 0.1% triton X-100 (PBST), followed by 2 x 10 min washes in 0.1 M Tris buffer. The slices (protected from light) were incubated in 320 µl distilled water, 25 µl 2 M Tris buffer, 50 µl 10 mM copper sulphate, 5 µl Azide ^594^ and100 µl 0.5 M ascorbic acid, for 30 minutes, then washed in Tris buffer ( 2 x 10 min).

*In vivo application of cholinergic drugs*

Mice (age 6-8 weeks) were injected intraperitoneally with EdU (0.1 ml at 10 mM) and either PNU 120596 (0.1 ml; 10 mM, number of animals = 4) or saline (0.1 ml) daily for a period of 4 days. Mice were anaesthetised and perfused with 4% PFA as above. 50 µm sections of thoracic (T4-8) and lumbar (L1-4) spinal cord were cut on a vibrating microtome. Sections were processed for EdU as above, except biotinylated azide was used and detected with Streptavidin Alexa^555^ (1:1000 in PBS + 0.1% Triton, Invitrogen).

*Immunohistochemistry for cultures and in vivo studies*

Immunofluorescence was performed with antibodies against PanQKI (mouse, Neuromab, 1:2), GFAP (mouse, Neuromab 1:100), CD24 (rat, BD Pharmingen 1:500), α1 Na+/K+ ATPase (NKAα1;rabbit, Epitomics, 1:1000)Tuj1 (chicken 1:500 Neuromics), Sox2 (goat 1:1000 Santa-Cruz), NeuN (mouse, Millipore, 1:1000). Antibodies were detected with appropriate Alexa ^488^ conjugated secondary antibodies. Slides were mounted for microscopy as above.

*Image capture and manipulation*

Sections were imaged using a Zeiss LSM510 Meta laser scanning confocal microscope equipped with both argon (λex=488 nm) and He-Ne (λex=543 nm) lasers and a 40x Fluor oil immersion objective. Images were captured using Carl Zeiss LSM software and images adjusted for brightness, contrast and intensity using CorelDraw16 software. Figures shown are single plane confocal images.

*Analysis of data*

For electrophysiology, change in membrane potential was recorded following ACh application and all data expressed as mean ± standard error (S.E.). Drug effects were determined using paired t-tests. When the CSFcCs were separated into different subtypes, one-way ANOVAs with post-hoc Bonferroni tests were used to determine the effects of ACh and two-way ANOVAs with post-hoc Bonferroni tests were used to determine the effects of cholinergic modulators on the ACh responses (n = no. of cells).

EdU positive cells were counted in the CC region (within 10 µm of the abluminal edge of the ependymal cells) directly through visualisation down a microscope using x 40 magnification. Cells were counted and checked by 2 investigators. In the cultured slices, to enable every cell throughout the thickness of the slice to be counted, the central canal region was divided into 4 quadrants and each quadrant counted in turn, focussing down through that quadrant. Using the same principle used for blood counts, any cells on the border between the quadrants were counted in for that quadrant if it was to the top or to the left and out for that quadrant if it was to the bottom or the right, thus eliminating the possibility of counting the same cell twice. Counts are given as the mean number of EdU positive cells per 300 µm slice in cultured slices and as mean number of EdU positive cells per 50 µm section for *in vivo* treatment (n = no. of slices or sections, N = no. of animals). All data are expressed as means ± S.E. and for statistical analysis, one-way ANOVAs with post-hoc Bonferroni tests determined differences in the numbers of proliferating cells between control and PNU treated slices.

1. Corns L, Deuchars J, Deuchars SA. GABAergic responses of mammalian ependymal cells in the central canal neurogenic niche of the postnatal spinal cord **Neurosci Lett***.* 2013;In press.
